# Bilateral cervical plexus block for anterior cervical spine surgery: study protocol for a randomised placebo-controlled trial

**DOI:** 10.1186/s13063-021-05377-4

**Published:** 2021-06-29

**Authors:** Michael J. Mulcahy, Thananchayan Elalingam, Kevin Jang, Mario D’Souza, Matthew Tait

**Affiliations:** 1 Department of Neurosurgery, Nepean Public Hospital, Sydney, Australia; 2 Macquarie Neurosurgery, Suite 201, 2 Technology Place, Sydney, Australia; 3 Department of Clinical Medicine, Faculty of Medicine and Health Sciences, Macquarie University, Sydney, Australia; 4 Central Clinical School, University of Sydney, Sydney, Australia

**Keywords:** Superficial cervical plexus, Cervical spine surgery, Randomised controlled trial, Placebo

## Abstract

**Background:**

There has been increasing focus to improve the quality of recovery following anterior cervical spine surgery (ACSS). Postoperative pain and nausea are the most common reasons for prolonged hospital stay and readmission after ACSS. Superficial cervical plexus block (SCPB) provides site-specific analgesia with minimal side effects, thereby improving the quality of recovery. The aim of our study was to investigate the effect bilateral cervical plexus block has on postoperative recovery in patients undergoing ACSS.

**Methods:**

The study is a pragmatic, multi-centre, blinded, parallel-group, randomised placebo-controlled trial. 136 eligible patients (68 in each group) undergoing ACSS will be included. Patients randomised to the intervention group will have a SCPB administered under ultrasound guidance with a local anaesthetic solution (0.2% ropivacaine, 15mL); patients randomised to the placebo group will be injected in an identical manner with a saline solution. The primary outcome is the 40-item quality of recovery questionnaire score at 24 h after surgery. In addition, comparisons between groups will be made for a 24-h opioid usage and length of hospital stay. Neck pain intensity will be quantified using the numeric rating scale at 1, 3, 6 and at 24 h postoperatively. Incidence of nausea, vomiting, dysphagia or hoarseness in the first 24 h after surgery will also be measured.

**Discussion:**

By conducting a blinded placebo trial, we aim to control for the bias inherently associated with a tangible medical intervention and show the true treatment effect of SCPB in ACSS. A statistically significant result will indicate an overall improved quality of recovery for patients; alternatively, if no benefit is shown, this trial will provide evidence that this intervention is unnecessary.

**Trial registration:**

ClinicalTrials.gov ACTRN12619000028101. Prospectively registered on 11 January 2019 with Australia New Zealand Clinical Trials Registry

## Background

Anterior cervical discectomy and fusion (ACDF) is a common surgical procedure for cervical spine disease. There has been an increasing focus on reducing the length of hospital stay after this procedure. Whilst the mean length of stay has been reported to be approximately 2 days, some centres are performing this procedure in an outpatient setting [1–4]. The approach for anterior cervical disc arthroplasty is the same for ACDF, and there is a trend towards performing this procedure in the outpatient setting too [5]. We will hereafter refer to these two operations under the heading anterior cervical spine surgery (ACSS).

Postoperative pain and nausea are the most common reasons for prolonged hospital stay and readmission after ACSS. [6, 7] Pre-emptive analgesia with regional anaesthesia is a method used to improve postoperative pain management. The superficial cervical plexus block (SCPB) has been used for neck surgeries for this reason [8]. The cervical plexus is formed by the anterior rami of the upper four cervical nerves and lies deep to the prevertebral fascia on the scalenus medius. In a SCPB, local anaesthesia is infiltrated at the punctum nervosum, anaesthetising four superficial branches of the cervical plexus: the lesser occipital, the great auricular, the transverse cervical, and the supraclavicular nerves. The distribution of these nerves includes the anterior and lateral neck, and the pre- and post-auricular areas.

SCPB is a safe technique that has been shown to provide good relief for incisional pain after thyroid and carotid surgeries, and relief of occipitonuchal pain after neurosurgical procedures [8–11]. It may also reduce opioid consumption and therefore the related side effects, such as nausea, vomiting, and respiratory depression. Finally, a goal of the SCPB is to improve the overall quality of recovery from surgery from the patient’s perspective.

The aim of this study is to investigate the effect bilateral cervical plexus block with local anaesthesia has on postoperative recovery in patients undergoing ACSS. The hypothesis is that bilateral SCPB is superior to placebo in patients undergoing ACSS in improving post-operative recovery, as measured by the 40-item quality of recovery (QoR-40) score at 24 h.

## Methods

### Design

This is a protocol for a pragmatic, multi-centre, blinded, parallel-group, randomised controlled placebo trial. The trial is being conducted in two metropolitan academic hospitals in Sydney, Australia (Macquarie University Hospital and Nepean Public Hospital).

All patients will undergo general anaesthesia (GA). The patients randomised to the intervention group will have a SCPB administered under ultrasound guidance with a local anaesthetic solution, whilst patients randomised to the placebo group will be injected with a saline solution. All patients will be admitted to the high dependency unit (HDU) post-operatively, which is routine practice in the primary centre.

**Fig. 1:**
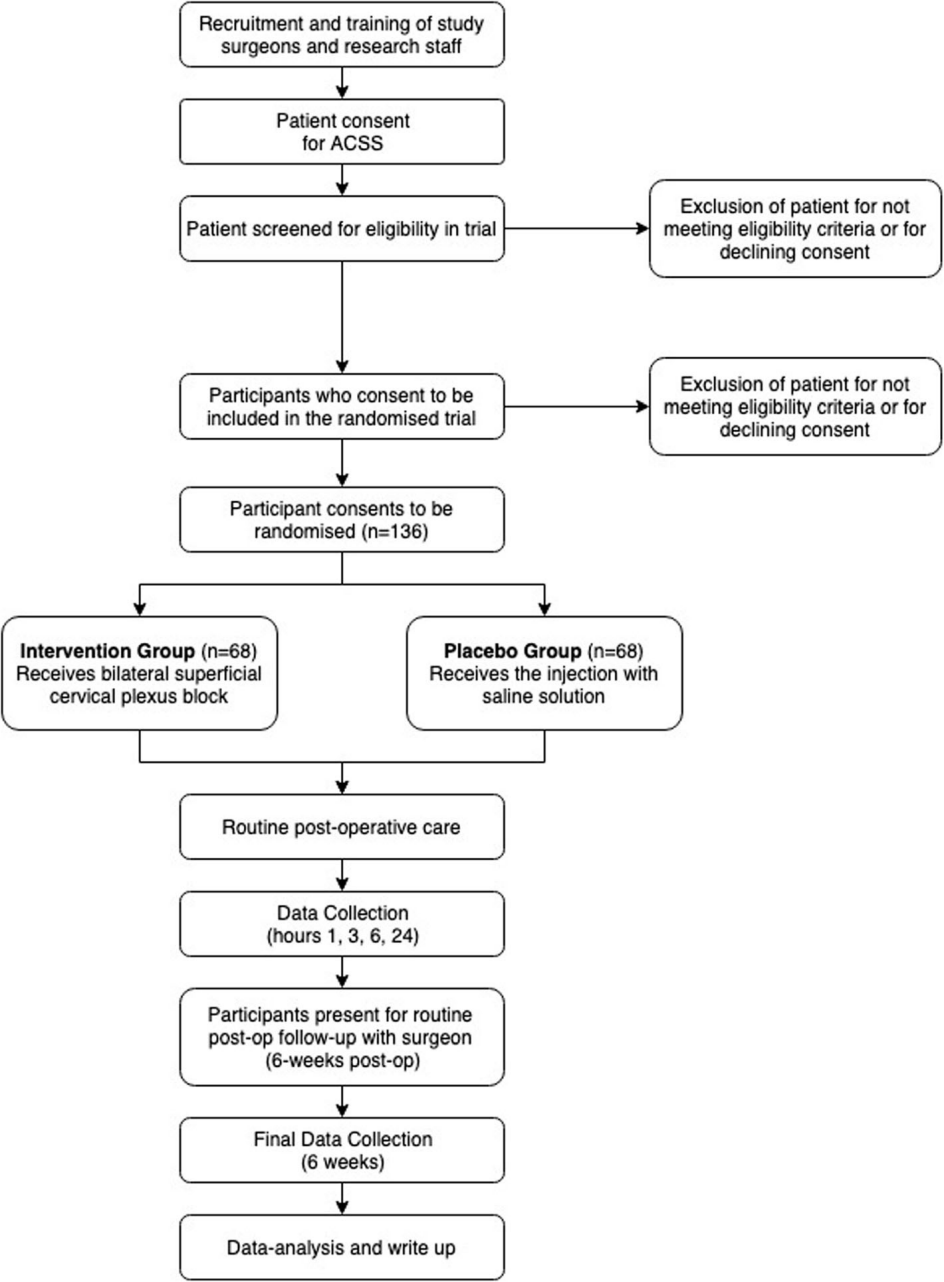
Participant timeline

The primary endpoint is the QoR-40 score at 24 h after surgery. It will be measured by blinded assessors. A participant timeline can be found in Fig. 1.

The trial design is pragmatic to focus on real-world patient-orientated outcomes, rather than trying to measure efficacy in an ideal scenario. As such, the inclusion criteria are based on the type of surgery being performed rather than the indication for surgery. Also, the retractor system and interbody devices used were not pre-specified, but based on the surgeon’s preference.

The trial was prospectively registered with the Australian New Zealand Clinical Trials Registry (ACTRN12619000028101).

### Study population and recruitment

Consecutive patients who present to one of the trial neurosurgeons rooms, hospital clinic or hospital emergency department will be assessed for eligibility after they have been consented to undergo ACSS. There are no financial incentives to patients or investigators to encourage enrolment. The eligibility criteria are listed below.

Inclusion criteria:

- Age ≥ 16 years old
- Undergoing anterior cervical discectomy and fusion
- Undergoing anterior cervical disc arthroplasty
- Isolated cervical spine trauma requiring anterior fusion only

Exclusion criteria:

- Multi-trauma
- Undergoing anterior cervical vertebrectomy and reconstruction
- Undergoing posterior fixation in addition to anterior surgery
- Undergoing surgery for malignancy
- Allergy to ropivacaine or bupivacaine
- Pregnant patients
- Neurologic or psychiatric condition that would prevent completion of the QoR-40 questionnaire

If the patient meets the eligibility criteria, they will be provided with a patient information and consent form, and be given time to read it, before undergoing an informed consent process with their treating neurosurgeon. All patients will receive a study enrolment number and baseline data will be obtained. Patients will be free to withdraw from the study at any time without giving a reason.

In addition to standard demographic information, other baseline data collected will include smoking history, pre-operative opioid use, adjuvant analgesia use, indication for ACSS and history of previous spine surgery.

### Intervention

All patients will receive standardised monitoring and one of two standardised anaesthetic regimens at the discretion of the primary anaesthetist.

Option 1: Induction with intravenous fentanyl, propofol and a non-depolarising muscle relaxant to facilitate intubation followed by maintenance with oxygen, air and sevoflurane.

Option 2: Induction using a total intravenous anaesthetic technique using Propofol and Remifentanil and infusions and a non-depolarising muscle relaxant and then maintenance with Propofol and Remifentanil infusions. This is usually indicated in patients where volatile anaesthesia may interfere with intra-operative neuromonitoring such as motor and sensory evoked potentials.

With either standardised regime, immediately after induction of anaesthesia, bilateral ultrasound-guided SCPB is placed by an anaesthetist experienced in delivering the block. Patients randomised to the local anaesthetic group will receive 15mL of 0.2% ropivacaine on each side (totaling 30mLs); the placebo group will receive 15mLs of 0.9% saline on each side. The cervical plexus is composed of purely sensory nerves, so a low concentration local anaesthetic was chosen to reduce the risk of local anaesthetic toxicity and inadvertent motor block [12].

All patients will receive skin infiltration at the incision site of 5mls of 0.25% bupivicaine with 1:400,000 adrenaline by the surgeon prior to incision, a local standard of care.

The technique for the SCPB described below is similar to the method described by the New York Society of Regional Anaesthesia for ultrasound-guided SCPB [13].

After the patient is positioned and the skin prepared with a chlorhexidine-alcohol scrub, the transducer is placed on the neck, overlying the sternocleidomastoid muscle (SCM) at its midpoint (approximately the level of the cricoid cartilage). Once the SCM is identified, the transducer is moved posteriorly until the tapering posterior edge is positioned in the middle of the screen. At this point, the brachial plexus and/or the interscalene groove (between the anterior and middle scalene muscles) should be identified. Then, the needle is passed through the skin, platysma and investing layer of fascia, placing the tip adjacent to the superficial cervical plexus. 15mL of the solution is administered to envelop the plexus.

Intraoperatively, all patients will receive 4mg of intravenous (IV) dexamethasone after induction as prophylaxis for postoperative nausea and vomiting (PONV) and may prolong the duration of the block anaesthesia and before surgical incision. Approaching closure of the wound, patients will receive 1g of IV paracetamol followed by 4mg of IV ondansetron or 1mg or IV granisetron for PONV prophylaxis.

Additional analgesia will be provided with incremental boluses of opioids at the discretion of the anaesthetist. At the end of the surgery, maintenance of anaesthesia either sevoflurane or propofol and remifentanil will be turned off and the neuromuscular blockade will be reversed with neostigmine (50 mg/kg IV) and glycopyrrolate (10 mcg/kg IV) or sugammadex.

Patients will then be taken to the post-anaesthetic care unit and then to the HDU, or directly to the HDU. The general post-operative care of the patients will be directed by the local standard practice in terms of monitoring and assessment of neurological status, pain, PONV and level of sedation.

Patients will be asked to rate their pain upon arrival and at regular intervals using an 11 point Numerical Rating Scale by the recovery nurse (0 = no pain to 10 = the worst pain imaginable; D= difficult to assess, U= unconscious). This score is recorded separately when at rest and with patient movement. The character of pain will be recorded as one of five choices (dull, pressure, radiating, sharp, throbbing). Nausea and vomiting will be assessed using a scoring system (0= no nausea 1= occasional nausea, 3= nausea and occasional vomiting, 4= constant vomiting). The level of sedation will be recorded using an analogue score (0= wide awake, 1= easy to rouse, 2= drowsy, 3= difficult to rouse, 4= unrousable). To maintain a pain score of <4, or the patient being clinically comfortable, an opioid will be administered IV every 2 to 5 min as needed. The choice of opioids will be at the discretion of the anaesthetist.

Difficulty with postoperative nausea and vomiting will be treated with 0.5mg of IV droperidol and/or 4mg of IV Ondansetron. In the first post-operative day, patients will receive regular paracetamol (1g four times a day) and oral opiates or a patient controlled analgesia (PCA), at the discretion of the anaesthetist.

### Randomisation

A patient will be randomised to the study once they have met the eligibility criteria. The randomisation of participants will occur via a central computer generated randomisation service—the Macquarie Clinical Trials Unit (who will not be involved in patient care, data collection or analysis). The day prior to surgery, the pharmacy will receive the randomisation code via email, allocating the patient to one of the two groups.

Due to expected differences in dissection technique, stratified randomisation will be performed based on the number of levels operated on (dichotomised to ≤2 or >2).

### Concealment

The hospital pharmacy will produce the solution after receiving the randomisation notice and will send the blinded solution to theatre on the day of surgery in a vial marked “Ropivacaine 0.2% OR Placebo 15mL” along with the patient’s name, study enrolment number and expiry date.

### Blinding

The surgeon and anaesthetist will be blinded to the solution being injected. The surgeon performing the operation will follow-up with the patient 6 weeks later and will remain blinded to their study allocation. The patient will not be aware which solution they will be receiving and will remain blinded to the study arm they are allocated to after the operation. The investigators conducting the questionnaires will not be aware of the treatment given to the patient.

### Primary outcome

The primary outcome will be the QoR-40 score measured at 24 h post-operation completion time. The ‘quality of recovery’ score was developed to provide a patient-orientated assessment of the quality of recovery after surgery and anaesthesia [14]. This was later expanded to the QoR-40 score. This score was validated and shown to be reliable when completed on the first post-operative day [15]. The score measures five dimensions of patient experience: pain, emotional state, physical independence, comfort and psychological support. A systematic review showed that the QoR-40 was a widely used, valid, reliable and responsive tool for assessing quality of recovery from a patient perspective [16].

The minimum clinically important difference (MCID) can be thought of as the smallest change that is important to a patient and is an especially important concept in studies involving patient-reported outcomes [17]. There are various methods of determining MCID. A 2016 study, using the anchor method, determined the MCID to be 6.3 for the QoR-40 score [18]. Based on cardiac studies, one author argued that a change of 5–7 signifies a clinically important difference in the QoR-40 score [19]. A randomised study of patients undergoing cervical spine surgery predefined the MCID as a change of 10 [20]. In a prospective study of patients undergoing neurosurgery procedures, the mean QoR-40 at post-operative day 1 in 92 patients undergoing spinal surgery was 160 (SD-15) [21]. Based on the distribution-based approach of determining MCID (that is, half standard deviation), the MCID would be 7.5. Taking this into account, we chose a change of 7.5 to signify a clinically important change in a spinal surgery setting.

### Secondary outcomes

- A 24-h opioid usage (converted to equivalent units of oral morphine).
- Numeric pain rating scale for neck pain at 1 h, 3 h, 6 h and at 24 h post-operative time.
- Incidence of nausea and vomiting, in the first 24 h after surgery.
- Hospital length of stay.

### Sample size calculation

The primary endpoint is the QoR-40 score at 24 h post-operatively, and thus, we are planning a study of a continuous response variable from independent control and experimental subjects with 1 control per group. We used an MCID of 7.5 for the QoR-40, as discussed above. If the true difference between the groups is 7.5, we will need a study of 64 subjects in each group to be able to reject the null hypothesis that the population means of the experimental and control groups are equal with probability (power) 0.8. The type 1 error associated with this test of the null hypothesis is 0.05.

Allowing for a dropout rate of 5%, we will randomise 136 patients (68 in each group). The software used to do the calculation was PS from the University of Vanderbilt, USA.

### Statistical analysis

Baseline characteristics will be summarised by treatment group to assess comparability. We are not planning to adjust the analyses for baseline variables.

Data analysis will be performed according to the intention-to-treat principle. The primary outcome will be analysed by a comparison of scores using an independent t test, with 95% confidence intervals and two-tailed p values reported. Significance levels will be set at 0.05.

The 24-h opiate usage and hospital length of stay will by analyses using independent t tests with 95% confidence intervals and two-tailed p values reported.

Nausea and vomiting will be recorded as categorical variables and analysed using the Pearson chi-squared test.

For the analysis of pain scores across the four periods a linear regression analysis will be used with the dependent variable being the pain score and the independent variables being ‘period’, ‘group’ and ‘period*group’ interaction. This analysis will be conducted within the framework of Generalised Estimating Equations, which corrects the bias in the estimates of the SE caused by having multiple observations per person and thus correlation in the data. The output generated would be the difference between the groups across all periods (95% CI and p value) and the difference between the groups in each period (95% CI and p value).

### Data collection and storage

All forms will be completed in English. Data will be collected directly from patients after enrolment. All paper copies of collected data will be stored in a locked filing cabinet in the Chief Investigator’s office and will be identified by the patient’s study enrolment number. The database of patient’s names and enrolment numbers will be stored on a password protected hard drive, thus preserving the de-identification of the paper copies.

All data will be entered in to an electronic trial database stored on a password protected external hard drive that will remain in the office of the chief investigator. The QoR-40 questionnaire will be completed by the patient with an investigator 24 h after surgery. Patients will follow-up with their treating neurosurgeon 6 weeks after surgery, and final data collection will occur at this time.

The data analysis will be carried out by the associate investigators in the trial, using the de-identified patient data.

Information and data will be stored for 10 years and then be erased. The Chief Investigators only will have access to the filing cabinet and hard drive.

Deidentified individual participant data that underlies the results reported in this trial will be made available to researchers who provide a written request to the chief investigators within 10 years after the publication of the trial. The request must be methodologically sound, and the researchers will need to sign a data access agreement.

### Ethics and dissemination

Ethics approval has been obtained from the Macquarie University Human Research Ethics Committee (HREC) for the trial to commence at Macquarie University Hospital (Reference No:5201949827706) and from the Nepean Blue Mountains Local Health District HREC to commence at Nepean Public Hospital (Reference 2019/ETH10684).

Any modifications to the protocol will be submitted to the above aforementioned HRECs before implementation, and the protocol will be amended on the ANZCTR website.

All results arising from this trial will be presented at scientific meetings and published in peer-reviewed journals. In addition to publishing the protocol in a peer-reviewed journal, the draft protocol is available for public access on the Australian New Zealand Clinical Trials Registry website. There is no intention to use professional writers.

### Monitoring and safety

All adverse events will be recorded. When a serious adverse event occurs, it will be reported immediately to the Macquarie University HREC or the Nepean Blue Mountains Local Health District HREC. The chief investigator will review all documentation related to the event and provide a written report to the HREC. Annual reports to the HREC are planned. There will not be an interim analysis or data monitoring committee: this trial is anticipated to be of a relatively short duration, is evaluating low risk interventions, is not evaluating a new treatment and is not influenced by industry.

Adverse events related to the SCPB to be recorded are as follows:

- Allergic reaction to local anaesthetic solution
- Local anaesthetic toxicity
- Accidental intravascular injection of local anaesthetic
- SCPB site haematoma
- Upper limb paresis
- Horner’s syndrome
- Phrenic nerve palsy

Each adverse event has a less than 1% chance of occurring with this procedure. If life-threatening adverse event occurs in the intra-operative period, unblinding will occur when the anaesthetist for the case calls pharmacy to request emergency unblinding.

## Discussion

Here, we present the protocol for a randomised controlled trial evaluating the role of SCPB in ACSS. The trial is unique as it is the first placebo trial and the first completely blinded trial of its nature. This protocol conforms to the Standard Protocol Items: Recommendations for Interventional Trials [22].

Whilst SCPB has been studied in thyroid and carotid surgery, there is a paucity of evidence for performing SCPB for ACSS. A single centre, non-blinded, randomised controlled trial (RCT) comparing GA to awake surgery with bilateral deep and superficial cervical plexus blocks found a trend towards better post-operative pain control in the cervical plexus block group. The GA group however had better intra-operative haemodynamic stability and patient satisfaction scores [23]. It has been shown that SCPB is preferred to deep cervical plexus block, as the deep block is associated with a greater risk profile without conferring a significant benefit [10].

The only trial we are aware of studying SCPB in ACSS is an RCT comparing unilateral SCPB with no block in patients undergoing single or two-level ACDF [20]. The primary outcome was the QoR-40 score measured at 24 h post-surgery. The patients were blinded, but the surgeon and anaesthetist were not. It was a single-centre study that enrolled 23 patients to each group. The QoR-40 scores were significantly better in the SCPB group (179 [116-195] vs. 157 [97-196]) (median [interquartile range]).

The disadvantages of this trial include the small number of patients, the lack of investigator blinding and the lack of a placebo. The chosen MCID of 10 points for the QoR-40 score is not reflective of the current literature (see above). In addition, we believe a bilateral SCBP yields superior outcomes to a unilateral block as the surgical incision can sometimes reach, or cross, the midline. Finally, the block was landmark-based, rather than ultrasound-guided. Ultrasound-guided SCPB has greater efficacy and reduced local anaesthetic toxicity compared to the landmark technique [24, 25]. The design of our proposed trial addresses each of these issues.

Placebo-controlled trials are particularly important when outcomes are based on patients’ subjective rating [26]. This trial is justified because there is a need to [1] demonstrate the true treatment effect of this intervention, [2] control for the bias that is associated with the tangible intervention and [3] provide a quantification of the adverse events of this intervention.

The anticipated benefits of SCPB in ACSS include improved post-operative pain control, resulting in a reduced length of hospital stay, decreased opioid requirements and an improved patient experience. Alternatively, this placebo-controlled trial may demonstrate no benefit from SCPB, and in that case, an unnecessary percutaneous procedure and administration of a medication will be avoided.

Potential limitations of this trial include a possible slow rate of recruitment secondary to patient concern over participating in an interventional placebo trial. This is a well-known issue for placebo trials (in surgery in particular); however, these trials are important and have been shown to be feasible [27, 28]. Second, we have chosen a power of 80% in our sample size calculation. Whilst this could be regarded as low power, it is consistent with conventional values; although we do acknowledge the possibility of type II error if the effect size is not as strong as predicted [29].

### Trial status

The trial is in the recruitment stage. The first patient was enrolled on October 21, 2019. Seventeen patients have been recruited to date. This is protocol version 11 (11/6/21). Recruitment is anticipated to be completed in October 2022.

## Data Availability

Not applicable.

## Abbreviations

ACDF: Anterior cervical discectomy and fusion
ACSS: Anterior cervical spine surgery
GA: General anaesthesia
HDU: High dependency unit
HREC: Human Research Ethics Committee
MCID: Minimum clinically important difference
PONV: Post-operative nausea and vomiting
RCT: Randomised controlled trial
SCPB: Superficial cervical plexus block
QoR-40: 40-item quality of recovery

## References

[1] Basques BA, Bohl DD, Golinvaux NS, Gruskay JA, Grauer JN (2014). Preoperative factors affecting length of stay after elective anterior cervical discectomy and fusion with and without corpectomy: a multivariate analysis of an academic center cohort. Spine (Phila Pa 1976)..

[2] Adamson T, Godil SS, Mehrlich M, Mendenhall S, Asher AL, McGirt MJ (2016). Anterior cervical discectomy and fusion in the outpatient ambulatory surgery setting compared with the inpatient hospital setting: analysis of 1000 consecutive cases. J Neurosurg Spine..

[3] Gruskay JA, Fu M, Basques BA, Bohl DD, Buerba RA, Webb ML, Grauer JN (2016). Factors affecting length of stay and complications after elective anterior cervical discectomy and fusion: a study of 2164 patients from The American College of Surgeons National Surgical Quality Improvement Project Database (ACS NSQIP). Clin Spine Surg..

[4] Fu MC, Gruskay JA, Samuel AM, Sheha ED, Derman PB, Iyer S, Grauer JN, Albert TJ (2017). Outpatient anterior cervical discectomy and fusion is associated with fewer short-term complications in one- and two-level cases: a propensity-adjusted analysis. Spine (Phila Pa 1976)..

[5] Mohandas A, Summa C, Worthington WB, Lerner J, Foley KT, Bohinski RJ, Lanford GB, Holden C, Wohns RNW (2017). Best practices for outpatient anterior cervical surgery: results from a delphi panel. Spine (Phila Pa 1976)..

[6] Garringer SM, Sasso RC (2010). Safety of anterior cervical discectomy and fusion performed as outpatient surgery. J Spinal Disord Tech..

[7] Arnold PM, Rice LR, Anderson KK, McMahon JK, Connelly LM, Norvell DC (2011). Factors affecting hospital length of stay following anterior cervical discectomy and fusion. Evid Based Spine Care J..

[8] Mayhew D, Sahgal N, Khirwadkar R, Hunter JM, Banerjee A (2018). Analgesic efficacy of bilateral superficial cervical plexus block for thyroid surgery: meta-analysis and systematic review. Br J Anaesth..

[9] Niijima K, Malis LI (1993). Preventive superficial cervical plexus block for postoperative cervicocephalic pain in neurosurgery. Neurol Med Chir (Tokyo)..

[10] Pandit JJ, Satya-Krishna R, Gration P (2007). Superficial or deep cervical plexus block for carotid endarterectomy: a systematic review of complications. Br J Anaesth..

[11] Egan RJ, Hopkins JC, Beamish AJ, Shah R, Edwards AG, Morgan JD (2013). Randomized clinical trial of intraoperative superficial cervical plexus block versus incisional local anaesthesia in thyroid and parathyroid surgery. Br J Surg..

[12] Narouze S (2009). Sonoanatomy of the cervical spinal nerve roots: implications for brachial plexus block. Reg Anesth Pain Med..

[13] NYSORA (New York School of Regional Anesthesia). Ultrasound-guided superficial cervical plexus block. New Jersey: NYSORA (New York School of Regional Anesthesia); 2018 [cited 2018 June 17] Available from: https://www.nysora.com/ultrasound-guided-superficial-cervical-plexus-block.

[14] Myles PS, Hunt JO, Nightingale CE, Fletcher H, Beh T, Tanil D, Nagy A, Rubinstein A, Ponsford JL (1999). Development and psychometric testing of a quality of recovery score after general anesthesia and surgery in adults. Anesth Analg..

[15] Myles PS, Weitkamp B, Jones K, Melick J, Hensen S (2000). Validity and reliability of a postoperative quality of recovery score: the QoR-40. British Journal of Anaesthesia..

[16] Gornall BF, Myles PS, Smith CL, Burke JA, Leslie K, Pereira MJ, Bost JE, Kluivers KB, Nilsson UG, Tanaka Y, Forbes A (2013). Measurement of quality of recovery using the QoR-40: a quantitative systematic review. British Journal of Anaesthesia..

[17] Copay AG, Subach BR, Glassman SD, Polly DW, Schuler TC (2007). Understanding the minimum clinically important difference: a review of concepts and methods. Spine J..

[18] Myles PS, Myles DB, Galagher W, Chew C, MacDonald N, Dennis A (2016). Minimal clinically important difference for three quality of recovery scales. Anesthesiology..

[19] Myles PS (2016). Clinically important difference in quality of recovery scores. Anesth Analg..

[20] Mariappan R, Mehta J, Massicotte E, Nagappa M, Manninen P, Venkatraghavan L (2015). Effect of superficial cervical plexus block on postoperative quality of recovery after anterior cervical discectomy and fusion: a randomized controlled trial. Can J Anaesth..

[21] Leslie K, Troedel S, Irwin K, Pearce F, Ugoni A, Gillies R, Pemberton E, Dharmage S (2003). Quality of recovery from anesthesia in neurosurgical patients. Anesthesiology..

[22] Chan AW, Tetzlaff JM, Altman DG, Laupacis A, Gøtzsche PC, Krleža-Jerić K, Hróbjartsson A, Mann H, Dickersin K, Berlin JA, Doré CJ, Parulekar WR, Summerskill WSM, Groves T, Schulz KF, Sox HC, Rockhold FW, Rennie D, Moher D (2013). SPIRIT 2013 statement: defining standard protocol items for clinical trials. Ann Intern Med..

[23] Wang H, Ma L, Yang D, Wang T, Wang Q, Zhang L, Ding W (2017). Cervical plexus anesthesia versus general anesthesia for anterior cervical discectomy and fusion surgery: a randomized clinical trial. Medicine (Baltimore)..

[24] Barrington MJ, Kluger R (2013). Ultrasound guidance reduces the risk of local anesthetic systemic toxicity following peripheral nerve blockade. Reg Anesth Pain Med..

[25] Senapathi TGA, Widnyana IMG, Aribawa I, Wiryana M, Sinardja IK, Nada IKW (2017). Ultrasound-guided bilateral superficial cervical plexus block is more effective than landmark technique for reducing pain from thyroidectomy. J Pain Res..

[26] Savulescu J, Wartolowska K, Carr A (2016). Randomised placebo-controlled trials of surgery: ethical analysis and guidelines. J Med Ethics..

[27] Hare KB, Lohmander LS, Roos EM (2014). The challenge of recruiting patients into a placebo-controlled surgical trial. Trials..

[28] Wartolowska K, Collins GS, Hopewell S, Judge A, Dean BJ, Rombach I (2016). Feasibility of surgical randomised controlled trials with a placebo arm: a systematic review. BMJ Open..

[29] Norman G, Monteiro S, Salama S (2012). Sample size calculations: should the emperor’s clothes be off the peg or made to measure?. BMJ..

